# Iron deficiency parameters in autism spectrum disorder: clinical correlates and associated factors

**DOI:** 10.1186/s13052-017-0407-3

**Published:** 2017-09-21

**Authors:** Serkan Gunes, Ozalp Ekinci, Tanju Celik

**Affiliations:** 1 Department of Child and Adolescent Psychiatry, Hatay State Hospital, Hatay, Turkey; 2 Department of Child and Adolescent Psychiatry, University of Health Sciences Medical Faculty, Istanbul, Turkey; 3 Department of Pediatrics, Dr. Behçet Uz Research and Training Hospital, Izmir, Turkey

**Keywords:** Anemia, Autism spectrum disorder, Ferritin, Hemoglobin, Iron

## Abstract

**Background:**

High prevalence of iron deficiency (ID) and iron deficiency anemia (IDA) has been reported in children with autism spectrum disorder (ASD). However, there is a limited number of studies about the association between iron deficiency parameters and clinical symptoms of ASD. This study aims to compare hemoglobin, hematocrit, iron, ferritin, MCV, and RDW levels between ASD patients and healthy controls and to investigate the correlation between these values and clinical symptoms of ASD.

**Methods:**

The sample consisted of 100 children in ASD patient group and 100 healthy controls, with an age range of 2–18 years. We used ferritin cutoff of < 10 ng/mL for preschoolers (< 6 years) and < 12 ng/mL for school-aged (> 6 years) children to evaluate ID. Anemia was defined as hemoglobin < 11.0 g/dL for preschoolers and < 12.0 g/dL for school-aged children. Childhood Autism Rating Scale (CARS), Autism Behavior Checklist (AuBC), and Aberrant Behavior Checklist (AbBC) were used to evaluate the severity of autistic symptoms and behavioral problems. Categorical variables were compared by using chi-square test. Normally distributed parametric variables were compared between groups by using Independent Samples t test. Pearson’s correlation analysis was used in order to examine the correlations. The *p* value < 0.05 was accepted to be statistically significant.

**Results:**

Hemoglobin, hematocrit, iron, and MCV (*p* < 0.05) levels of children with ASD were lower than healthy controls. Hemoglobin, hematocrit, and MCV (*p* < 0.05) levels were found to be significantly lower in preschool ASD patients. Hemoglobin and hematocrit (*p* < 0.05) levels were significantly lower in ASD patients with intellectual disability. Hemoglobin (*p* < 0.05) levels were lower in patients with severe ASD. There was a significant negative correlation between hematocrit levels of children with ASD and CARS, AuBC, and AbBC total scores (*p* < 0.05).

**Conclusions:**

Hemoglobin levels of children with ASD were lower than healthy children, but this was not sufficient to result in anemia. IDA in children with ASD might be associated with intellectual disability instead of ASD symptom severity.

## Background

Autism spectrum disorder (ASD) represents a group of disorders characterized by impairment in three developmental areas: social interaction, communication, and restricted and repetitive behaviors [1–3]. High prevalence of iron deficiency (ID) and iron deficiency anemia (IDA) was reported in children with ASD [4–6]. Inadequate iron intake and malabsorption were thought to cause ID in these children [7–9]. Insufficient dietary iron intake was considered to be associated with food selectivity which is commonly seen in this population [3].

Iron plays an important role in cognitive, behavioral, and motor development [3, 4]. A reduction of iron levels in the brain may be accompanied by changes in serotonergic and dopaminergic systems, cortical networks, and myelination [10]. In ID, learning, attention, memory, and psychomotor functions may be affected due to functional deficits in these biological processes [4]. Additionally, it is discussed that children with anemia may explore the environment less and move less than healthy children and anemia may prevent them from receiving sufficient stimulus and developing new skills [11]. Thus, it can be considered that ID/IDA may increase the severity of autistic symptoms in children with ASD.

There is limited literature about the association between iron deficiency parameters and clinical symptoms of ASD. So, this study aims to compare hemoglobin, hematocrit, iron, ferritin, MCV, and RDW levels of children with ASD with healthy controls and to investigate the clinical correlates and associated factors of ID and IDA in ASD.

## Methods

### Sample

The study sample was recruited from our child and adolescent psychiatry department between March 2014 and April 2015.

The inclusion criteria for the patient group were as follows: 1) ASD diagnosis according to DSM-V. 2) An age range of 2–18 years. 3) Not having an infection, inflammatory conditions, or chronic physical illness. 4) Not receiving iron supplementation and going on a diet. ASD diagnosis was made using the information gathered by history taking, clinical symptoms and observations, CARS, and checklists. One of the authors, who is a faculty member of the same hospital and has an extensive experience in ASD, reviewed all the data and confirmed the ASD diagnoses. Initially, 116 children with DSM-V ASD diagnosis were recruited. From these children, 16 were excluded for the following reasons: Six parents of children with ASD did not want to participate in the study, four children had a continuing infection, two children had a chronic medical illness, and four children were excluded by the faculty member for not meeting ASD diagnostic criteria. So, the final sample for the patient group included 100 children with ASD.

The inclusion criteria for the control group were as followings: 1) No psychiatric disorder diagnosis according to DSM-V 2) An age range of 2–18 years. 3) Not having an infection, inflammatory conditions, chronic physical or mental illness. 4) Not receiving iron supplementation and going on a diet. 5) For preschoolers, normal developmental history and normal Denver II Test. 6) For school-aged children, normal intelligence based on either a WISC-R full scale IQ score above 80 or the average/above academic functioning documented with the last year’s final school grades. For the control group, 100 children (an equal number with patients), who referred to the department for counseling about child development, school adjustment and performance, teenage problems, family and friend relations, were recruited.

### Intellectual evaluation

Intellectual capacities of children in both patient and control groups were examined. A child development specialist performed Denver II Test to children under 6 years. An experienced psychologist performed WISC-R full scale to children over 6 years. Children who could not adapt to Denver II Test and WISC-R full scale were evaluated with developmental history, clinical symptoms and observations, and the average academic functioning documented with the last year’s final school grades. In the patient group, children with normal intellectual capacity and border intellectual disability were accepted as normal and children with mild, moderate, and severe intellectual disability were accepted as intellectual disability. In the control group, 13 children were preschoolers, 72 children were school-aged children, and 15 children were over 16 years. Sixty-six of school-aged children were administered the WISC-R full scale and 6 children were not. For 21 children, normal intellectual capacity was established based on the average academic functioning documented with the last year’s final school grades.

### Instruments

#### Childhood autism rating scale

The Childhood Autism Rating Scale (CARS) consists of 15 items and all the items contribute equally to one total score. Each item is rated by half scoring between 1 and 4 [12]. 15–29 points represent the child does not have autism, 30–36.5 points represent mild-moderate autism, and 37–60 points represent severe autism. For the Turkish version of the scale, item analysis shows that all items (except item 14) differentiate children with mild to severe autism [13]. According to CARS total score, children with ASD were divided into two groups: children with mild-moderate ASD and children with severe ASD.

#### Autism behavior checklist

The Autism Behavior Checklist (AuBC) is especially used in determining the frequency and severity of autistic symptoms [14]. The checklist includes 57 questions divided into five categories. The validity and the reliability of AuBC were satisfying for the Turkish sample [15].

#### Aberrant behavior checklist

The Aberrant Behavior Checklist (AbBC) is useful for evaluating behavioral problems in children [4]. It consists of 58 items and each item is rated on a four-point scale ranging from 0 (not a problem) to 3 (the problem is severe). The Turkish translation and adaptation of the checklist were conducted by Sucuoglu [16] and Karabekiroglu and Aman [17].

### Laboratory measurements

Serum ferritin level was used as an indicator for ID since it is a precursor for ID and represents iron levels in body tissues including brain [4]. We used ferritin cutoff < 10 ng/mL for preschool children and < 12 ng/mL for school-aged children to evaluate ID [3, 5, 6]. Anemia was defined as hemoglobin < 11.0 g/dL for preschool children and < 12.0 g/dL for school-aged children [3]. The following cutoffs were used based on our hospital laboratory values: iron < 50 μg/dL, hematocrit < 32%, MCV < 75 fL, and RDW > 14.5%. Hemoglobin, hematocrit, iron, ferritin, MCV, and RDW values were measured in fasting blood in the morning at our hospital biochemistry laboratory using standard measurement assays.

### Statistical analysis

The collected data were analyzed by using the SPSS version 16.0 (SPSS, Inc., Chicago, IL, USA). Clinical data were shown as means and SD. Categorical variables were compared by using chi-square test. Normally distributed parametric variables were compared between groups by using Independent Samples t test. The Mann–Whitney U test was used to compare the age difference between ASD children and healthy controls. Pearson’s correlation analysis was used in order to examine the correlation between hemoglobin, hematocrit, iron, ferritin, MCV, RDW values and CARS, AuBC, and AbBC total scores. The *p* value < 0.05 was accepted to be statistically significant.

## Results

The sample consisted of 100 children in ASD patient group and 100 healthy controls, with an age range of 2–18 years (median (IQR) = 10 (6–13), mean ± SD = 9.73 ± 4.20). Mean age of the patient group was 8.36 ± 4.22 years, median age was 8 (4.25–11.75) years, and 84% (*N* = 84) were males. Control group has a mean age of 11.01 ± 3.73 years, median age of 11 (8–14) years, and 71% (*N* = 71) were males. The median age of healthy controls was significantly higher than that of ASD patients (*p* < 0.05). Fifty percent (*N* = 50) of the patients were mild-moderate ASD and 50% (N = 50) were severe ASD. Forty-two percent (*N* = 42) of the patient group had normal intellectual capacity and 58% (*N* = 58) had intellectual disability.

The prevalence of ID/IDA in patient and control groups is shown in Table 1. As seen in the table, although the prevalence of ID and IDA were higher in the patient group, no significant difference was found between the groups (*p* > 0.05). Table 1 also shows the comparison of hemoglobin, hematocrit, iron, ferritin, MCV, and RDW levels between patient and control groups. Hemoglobin, hematocrit, iron, and MCV (*p* < 0.05) levels were found to be lower in children with ASD.

**Table 1:** The prevalence of ID/IDA and the comparison of hemoglobin, hematocrit, iron, ferritin, MCV, and RDW levels between patient and control groups

	ASD patients, *N* = 100 N (%)	Controls, *N* = 100 N (%)	RR (95% CI)	p*
ID	25 (25.0)	15 (15.0)	1.66 (0.93–2.96)	0.077
IDA	13 (13.0)	6 (6.0)	2.16 (0.85–5.47)	0.091
	Mean (SD)	Mean (SD)	*d*	p**
Hemoglobin	12.68 (1.19)	13.02 (1.14)	0.291	0.045
Hematocrit	36.43 (4.61)	38.74 (5.78)	0.441	0.002
Iron	56.06 (31.98)	73.97 (33.85)	0.543	0.000
Ferritin	27.83 (22.01)	26.75 (17.65)	− 0.054	0.702
MCV	74.25 (8.46)	78.42 (5.41)	0.587	0.000
RDW	14.69 (5.78)	13.60 (1.09)	− 0.262	0.066

*RR* relative risk

*d* Cohen’s d

*Chi-Square test was used, **Independent-Samples t test was used

Table 2 shows the prevalence of ID/IDA between age groups in ASD patients. No significant difference was found between the groups (*p* > 0.05). Table 2 also shows the comparison of hemoglobin, hematocrit, iron, ferritin, MCV, and RDW levels between age groups. Hemoglobin, hematocrit, and MCV (*p* < 0.05) levels were found to be significantly lower in preschool ASD patients.

**Table 2:** The prevalence of ID/IDA and the comparison of hemoglobin, hematocrit, iron, ferritin, MCV, and RDW levels between age groups in ASD patients

	< 6 years, *N* = 46 N (%)	> 6 years, *N* = 54 N (%)	RR (95% CI)	p*
ID	13 (28.2)	12 (22.2)	1.27 (0.64–2.50)	0.487
IDA	5 (10.8)	8 (14.8)	0.73 (0.25–2.08)	0.559
	Mean (SD)	Mean (SD)	*d*	p**
Hemoglobin	12.18 (0.92)	13.06 (1.16)	0.840	0.000
Hematocrit	35.05 (2.45)	37.60 (5.63)	0.587	0.006
Iron	55.93 (27.62)	63.60 (30.06)	0.265	0.190
Ferritin	23.75 (17.33)	31.31 (24.96)	0.351	0.087
MCV	70.58 (9.99)	77.37 (5.21)	0.852	0.000
RDW	15.57 (8.41)	13.93 (1.11)	− 0.273	0.161

*RR* relative risk

*d* Cohen’s d

*Chi-Square test was used, **Independent-Samples t test was used

The comparison of hemoglobin, hematocrit, iron, ferritin, MCV, and RDW levels between ASD patients with normal intellectual capacity and intellectual disability is shown in Table 3. Hemoglobin and hematocrit (*p* < 0.05) levels were significantly lower in ASD patients with intellectual disability.

**Table 3:** The comparison of hemoglobin, hematocrit, iron, ferritin, MCV, and RDW levels between ASD patients with normal intellectual capacity and intellectual disability

	Normal intellectual capacity, *N* = 42 Mean (SD)	Intellectual disability, *N* = 58 Mean (SD)	*d*	*p*
Hemoglobin	12.97 (1.04)	12.42 (1.16)	0.499	0.017
Hematocrit	37.78 (3.05)	35.45 (5.29)	0.539	0.012
Iron	62.87 (29.87)	58.05 (28.57)	0.164	0.416
Ferritin	29.63 (27.61)	26.53 (16.99)	0.135	0.489
MCV	74.01 (10.52)	74.42 (6.66)	− 0.046	0.813
RDW	15.48 (8.85)	14.11 (0.99)	0.217	0.244

Independent-Samples t test was used

*d* Cohen’s d

Table 4 shows the comparison of hemoglobin, hematocrit, iron, ferritin, MCV, and RDW levels between patients with mild-moderate ASD and severe ASD. Hemoglobin (*p* < 0.05) levels were found to be significantly lower in patients with severe ASD.

**Table 4:** The comparison of hemoglobin, hematocrit, iron, ferritin, MCV, and RDW levels between patients with mild-moderate ASD and severe ASD

	Mild-moderate ASD, *N* = 50 Mean (SD)	Severe ASD, *N* = 50 Mean (SD)	*d*	*p*
Hemoglobin	12.91 (0.98)	12.39 (1.24)	0.465	0.024
Hematocrit	37.30 (3.09)	35.55 (5.65)	0.384	0.058
Iron	63.43 (28.21)	56.72 (29.82)	0.231	0.251
Ferritin	31.22 (26.11)	24.45 (16.53)	0.309	0.124
MCV	74.14 (9.65)	74.35 (7.16)	− 0.024	0.900
RDW	15.30 (8.11)	14.07 (1.03)	0.212	0.291

Independent-Samples t test was used

*d* Cohen’s d

Table 5 shows the evaluation of IDA in ASD patients according to intellectual capacity and ASD severity. The prevalence of IDA was found to be significantly higher in children with intellectual disability and severe ASD (*p* < 0.05).

**Table 5:** The evaluation of IDA in ASD patients according to intellectual capacity and ASD severity

		IDA (+) N (%)	IDA (−) N (%)	RR (95% CI)	*p*
Intellectual Capacity	Normal Intellectual Capacity *N* = 42	2 (4.7)	40 (95.3)	3.98 (0.93–17.03)	0.037
	Intellectual Disability *N* = 58	11 (18.9)	47 (81.1)		
ASD Severity	Mild-Moderate *N* = 50	1 (2.0)	49 (98.0)	12.00 (1.62–88.84)	0.001
	Severe *N* = 50	12 (24.0)	38 (76.0)		

Chi-Square test was used

*RR* relative risk

The correlations between hemoglobin, hematocrit, iron, ferritin, MCV, and RDW levels of ASD patients and CARS, AuBC, and AbBC total scores are shown in Table 6. As seen in the table, hematocrit was found to be negatively correlated with CARS, AuBC, and AbBC total scores (*p* < 0.05).

**Table 6:** The correlations between hemoglobin, hematocrit, iron, ferritin, MCV, and RDW levels of ASD patients and CARS, AuBC, and AbBC total scores

	CARS		AuBC		AbBC	
	*r*	*p*	*r*	*p*	*r*	*p*
Hemoglobin	− 0.133	0.188	− 0.109	0.289	− 0.027	0.796
Hematocrit	− 0.328	0.001	− 0.208	0.041	− 0.309	0.002
Iron	− 0.146	0.148	− 0.067	0.512	− 0.077	0.452
Ferritin	− 0.058	0.565	− 0.017	0.872	− 0.089	0.385
MCV	0.088	0.381	− 0.039	0.704	− 0.052	0.614
RDW	− 0.112	0.267	− 0.043	0.676	− 0.067	0.517

Pearson’s correlation analysis was used

*r* Pearson’s correlation coefficient

## Discussion

The association of ID/IDA and ASD has been the subject of studies and these studies have reported that ID/IDA is more common in children with ASD [3, 5, 6, 9]. In a study investigating iron levels in ASD patients between 19 months and 13 years, ID was seen in 52% of children with ASD [6]. Another study showed serum ferritin concentrations as lower than normal levels in 8.3% of autistic children between 1 and 2 years, 14.2% of autistic children between 3 and 5 years, and 20% of autistic children between 6 and 10 years [5]. Unlike this study, Herguner et al. [3] declared that ID was more common in preschool children with autism. In this study, 24.1% of autistic children had ID and 15.5% had IDA. Bilgiç et al. [4] reported that the prevalence of ID in autistic children under 6 years was higher than autistic children over 6 years and ID was found in 32% of preschool autistic children. Similar to Herguner’s study, 25% of children with ASD had ID and 13% of had IDA in our study. Hemoglobin, hematocrit, and MCV levels of preschool ASD patients were significantly lower than school-aged ASD patients. Dosman et al. [5] reported dietary iron intake in preschool children was two times more inadequate than school-aged children. Xia et al. [18] stated iron intake in autistic children was increased gradually between 2 and 9 years. Based on this information, it can be speculated that younger children with ASD may be more selective in nutrition and therefore ID is more common in this age group.

Previous studies about ID/IDA in ASD patients have no control groups [3–5]. But in our study, blood levels of children with ASD were compared with healthy controls. In this context, we found that serum hemoglobin, hematocrit, iron, and MCV levels of children with ASD were significantly lower than healthy children. Patient and control groups were compared in terms of ID/IDA and, although ID/IDA was higher in the patient group, it was not statistically significant. Based on these findings, it may be suggested that hemoglobin levels of children with ASD are lower than healthy children, but this is not sufficient to result in anemia. It should be kept in mind that half of our sample were children with mild-moderate ASD severity and this may be associated with the low frequency of IDA.

There is limited literature on the association between intellectual disability and ID/IDA [19]. ID/IDA has been found to be correlated with intellectual disability and cognitive impairments [20, 21]. To further investigate this association, we examined the blood levels and IDA of children with ASD according to intellectual capacity. Hemoglobin and hematocrit levels of our patients with normal intellectual capacity were significantly higher than those with intellectual disability. IDA was also found to be higher in ASD patients with intellectual disability. This finding may be related to the increased risk of feeding problems in intellectual disability. When compared with normal children, food selectivity, feeding skills deficits, food refusal and associated behavior problems are more common in children with intellectual disability [19].

Hemoglobin levels of children with mild-moderate ASD were significantly higher than children with severe ASD in our study. These two groups were examined in terms of IDA, and IDA was found to be higher in severe ASD group. IDA was seen in 2% of children with mild-moderate ASD and 24% of children with severe ASD. A study made in Turkey showed IDA was seen in 3.9% of school-aged children [22]. In another study conducted in Turkey, it was reported the prevalence of IDA in children was 5% [23]. According to these studies, the IDA prevalence of children with mild-moderate ASD in our study was similar to normal population. The IDA prevalence of children with severe ASD was too higher than normal population. On the other hand, the prevalence of IDA was higher in both children with severe ASD and children with intellectual disability in our study. It is known that intellectual disability is more serious in children with severe ASD than children with mild-moderate ASD [24]. Therefore, we suggest that IDA in children with ASD may be associated with intellectual disability instead of ASD symptom severity.

The possible association between serum ferritin levels and autistic symptomology has been studied in a few studies [4, 5, 9, 25]. In one study, a correlation between low serum ferritin levels and high Autism Diagnostic Observation Schedule (ADOS) communication domain scores was reported [5]. In another study, no correlation between serum ferritin levels and ADOS and Autism Diagnostic Interview-Revised (ADI-R) scores in preschool children was seen. But, an inverse correlation between ferritin levels and ADOS communication domain scores in school children was suggested [9]. A study conducted by Bilgiç et al. [4] reported there was no significant correlation between serum ferritin levels and autistic symptom scores. Liu X et al. also found no correlation between ferritin concentration and CARS scores [25]. In our study, we determined there was no significant correlation between hemoglobin, iron, ferritin, MCV, RDW levels and CARS and AuBC total scores. However, there was a significant negative correlation between hematocrit levels of children with ASD and CARS and AuBC total scores.

Negative effects of ID on brain development are widely accepted theory and it is expected behavioral problems in children with IDA are seen more frequently [21, 26, 27]. ID/IDA was showed to cause behavioral problems in children with no medical diseases in many studies. In these studies, behavioral problems caused from ID were reported to be associated with social interaction problems and learning disabilities [21, 26, 27]. However, all the studies made on this subject do not give the same results and there are also studies that indicate no relationship between ID/IDA and behavioral problems [4]. In our study, there was no correlation between hemoglobin, iron, ferritin, MCV, RDW levels and AbBC total scores. Interestingly, same as CARS and AuBC total scores, there was a significant negative correlation between hematocrit levels and AbBC total score. The presence of such a correlation only with hematocrit and the small effect size may suggest that this finding may be coincidental.

The results of the present study should be evaluated within the context of its limitations. The first issue is the cross-sectional nature of the study which does not allow investigating the causality between ID/IDA and ASD. A second limitation is the age difference between the patient and control groups. The ages of ASD children were significantly lower than healthy individuals. It should be kept in mind that lower blood level of ASD children than healthy controls may be partly related to the age difference. Thirdly, a food survey was not used to record the diet of children. Finally, we did not have an inflammation marker and may have missed some ID cases due to falsely elevated ferritin.

## Conclusions

We stated that hemoglobin levels of children with ASD were lower than healthy children, but this was not sufficient to result in anemia. Hemoglobin levels of children with severe ASD were lower than children with mild-moderate ASD and IDA was higher in severe ASD patients. Hemoglobin and hematocrit levels of ASD patients with intellectual disability were lower than ASD patients with normal intellectual capacity and IDA was found to be higher in children with intellectual disability. We also indicated that there was a significant correlation between hematocrit levels of children with ASD and CARS, AuBC, and AbBC total scores. But, future studies are needed to clarify this finding.

## Abbreviations

AbBC: Aberrant behavior checklist
ADI-R: Autism diagnostic interview-revised
ADOS: Autism diagnostic observation schedule
ASD: Autism spectrum disorder
AuBC: Autism behavior checklist
CARS: Childhood autism rating scale
DSM-V: Diagnostic and statistical manual of mental disorders, 5th edition
ID: Iron deficiency
IDA: Iron deficiency anemia
MCV: Mean corpuscular volume
RDW: Red cell distribution width
WISC-R: Wechsler intelligence scale for children-revised
